# Cross protective immune responses in nursing piglets infected with a US spike-insertion deletion porcine epidemic diarrhea virus strain and challenged with an original US PEDV strain

**DOI:** 10.1186/s13567-017-0469-7

**Published:** 2017-10-06

**Authors:** Thavamathi Annamalai, Chun-Ming Lin, Xiang Gao, Xinsheng Liu, Zhongyan Lu, Linda J. Saif, Qiuhong Wang

**Affiliations:** Food Animal Health Research Program, Ohio Agricultural Research and Development Center, Department of Veterinary Preventive Medicine, The Ohio State University, 1680 Madison Ave., Wooster, OH 44691 USA

## Abstract

We investigated cross-protective immunity of a US spike-insertion deletion porcine epidemic diarrhea virus (PEDV) Iowa106 (S-INDEL) strain against the original US PEDV (PC21A) strain in nursing piglets. Piglets were inoculated orally with S-INDEL, PC21A or mock. At 20–29 days post-inoculation (dpi), all pigs were challenged with the PC21A strain. The S-INDEL-inoculated pigs had lower ileal IgA antibody secreting cells, serum IgA and neutralizing antibody titers compared with PC21A-inoculated pigs. No pigs in the PC21A-group developed diarrhea, whereas 81 and 100% of pigs in the S-INDEL and mock-groups had diarrhea post challenge, respectively. S-INDEL induced partial protective immunity against the original US PEDV strain.

## Introduction, methods, and results

Porcine epidemic diarrhea virus (PEDV) belongs to the *Coronaviridae* family and causes severe gastroenteritis and high mortality in neonatal piglets [1]. Outbreaks of PEDV in the US starting from 2013 [2] resulted in estimated economic losses of $ 900 million [3]. PEDV outbreaks were reported in 36 states in the US by the National Animal Health Laboratory Network as of January, 2016 (https://www.aasv.org/Resources/PEDv/PEDvWhatsNew.php). PEDV transmission occurs mainly through the fecal–oral route.

Phylogenetic analysis revealed that the original US PEDV strains were closer to the emerging PEDV strain AH2012 from China than to the classical PEDV strains [4]. Apart from the original US PEDV strains, variants that contain insertions and deletions in the S1 subunit of the spike (S) protein similar to the classical PEDV strains have been identified in the US. They were designated as “S-INDEL” PEDV strains, likely resulting from multiple recombination events between the classical and emerging PEDV strains in Asia [4–6]. Infection with S-INDEL strain causes less severe infection and low mortality compared with the original highly virulent US PEDV strains [7, 8]. The spike protein is a membrane glycoprotein that plays a major role in virulence, receptor binding [9, 10], and induction of protective immunity during PEDV infection [11].

Similar to the immunization strategies to control transmissible gastroenteritis (TGE) infection [12], lactogenic immunity is important to reduce morbidity and mortality associated with PEDV infection in neonatal piglets. Specifically, secretory IgA antibodies in colostrum and milk play a critical role in conferring protective immunity against enteric viral infections in suckling piglets [13]. However, once lactogenic immunity is curtailed post-weaning, piglets become susceptible to PEDV. Thus active immunization is essential to reduce economic losses associated with PEDV infection in weaned piglets. Vaccination against PEDV was extensively implemented in South Korea [14] and China [15], but with little success after 2010. A lower effectiveness of the vaccines may be associated with the emergence of new variants of PEDV [15]. Therefore effective PEDV vaccines against the emerging PEDV strains are urgently needed, but not yet available. Recently, we reported the mild virulence and partial cross-protection of a US S-INDEL PEDV Iowa106 strain against the original US PEDV PC21A strain in nursing pigs [8]. In this study, systemic and local humoral immune responses were assessed after infection of piglets with an S-INDEL strain (Iowa106) and challenge with the original US PEDV strain PC21A.

**Figure 1:**
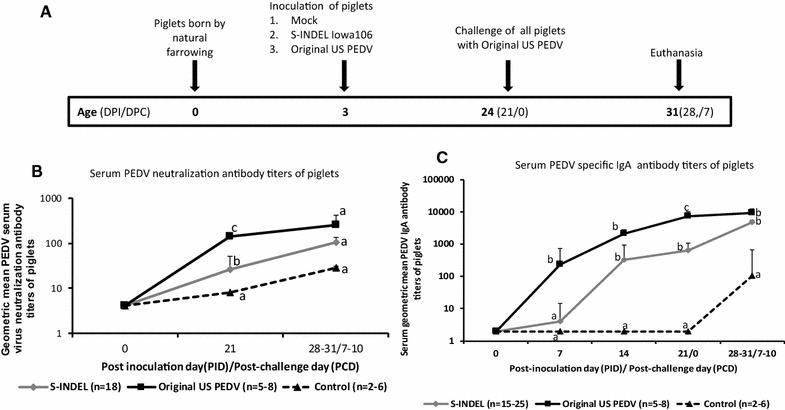
**Schematic diagram of the experimental design and serum PEDV specific antibody responses of piglets. A** Age of piglets (days), virus inoculation and challenge with original US PEDV strain were shown. Piglets were inoculated with the respective virus inoculum at 3–4 days of age and the remaining piglets were challenged with US PEDV strain PC21A at 20–29 days post-inoculation (dpi). **B** Piglet serum virus neutralization antibody titers were quantified by plaque reduction virus neutralization assay. **C** Piglet serum virus-specific-IgA antibody responses were measured by ELISA. Different alphabetical letters indicate significant differences (*p* < 0.05) at the same time point among groups, whereas the same letters indicate no significant difference. Dpi: day post-inoculation, dpc: day post-challenge.

All experiments were conducted in accordance with guidelines approved by the Institutional Animal Care and Use Committee (IACUC) at the Ohio State University. Virus inocula of the original US PEDV PC21A (GenBank accession no. KR078299) and S-INDEL PEDV Iowa106 (GenBank accession no. KJ645695) were prepared as described previously [8]. These two PEDV variants share 99% nucleotide identity at the genomic level. Six large white × Duroc crossbred pregnant animals were purchased from a specific pathogen free swine herd of The Ohio State University. The experimental design was described in detail in our earlier report [8] and is summarized in Figure 1A. Pig litters were randomly assigned to the following groups: (1) inoculated with S-INDEL Iowa106 and challenged with original US PEDV PC21A (four litters, *n* = 36); (2) inoculated with original US PEDV PC21A and challenged with the homologous strain (one litter, *n* = 11); and (3) mock inoculated and challenged with original US PEDV PC21A (one litter, *n* = 7). Piglets were inoculated with the respective virus inoculum at 3–4 days of age and a subset of pigs [S-INDEL (*n* = 8), original US PC21A (*n* = 3), control, (*n* = 1)] were euthanized at 2–3 weeks post inoculation. The remaining piglets were challenged with US PEDV strain PC21A at 20–29 days post-inoculation (dpi). Rectal swabs were collected to assess the severity of diarrhea and fecal virus shedding. Fecal consistency scores 0, 1, 2 and 3 correspond to normal, pasty, semi-liquid, and liquid feces, respectively, with scores of ≥ 2 corresponding to diarrhea. Virus RNA shedding was titrated by TaqMan real-time reverse transcription-PCR (RT-qPCR) as described previously [5, 8]. Serum samples were collected weekly from piglets and sows to assess PEDV specific antibody responses. Milk samples were also collected weekly from sows and whey was prepared from milk following protocols described earlier [19] to remove components (fat globules, casein micelles, and cells) that are known to interfere with immunological assays to determine virus specific antibody responses. All remaining piglets [S-INDEL (*n* = 16), original US PC21A (*n* = 4), control, (*n* = 5)] were euthanized at 7 days post-challenge (dpc) and ileal samples were collected to isolate mononuclear cells (MNCs) for enzyme-linked immunospot (ELISPOT) assays (see below) [16].

ELISA was developed for the detection of PEDV specific IgA antibodies. Hyperimmune anti-serum to PEDV used in the ELISA was produced in guinea pigs using repeated immunizations in a standard protocol approved by the IACUC at The Ohio State University. The virus used for immunization of guinea pigs and for ELISA was the tissue culture-adapted original US PEDV strain PC22A (GenBank Accession No. KM392224), which was isolated from the same PEDV outbreak as the PC21A strain [5]. The ELISA for the IgA antibody was adapted from previous standardized protocols [17, 18] with slight modifications. Nunc Maxisorp^®^ 96-well plates (Nunc-Immuno, Denmark) were coated with the guinea pig hyperimmune anti-serum against PEDV in coating buffer (bicarbonate/carbonate buffer, pH 9.6) at 37 °C for 2 h. The plates were washed with phosphate buffered saline-Tween (0.05%) (PBS-T) and blocked with 4% skim milk (Great Value™ instant dry milk) diluted in PBS-T overnight at 4 °C. After washing, virus/mock (PEDV-infected Vero cell supernatants/mock-infected Vero cell supernatants prepared as described previously [5]) were added to the wells and incubated at 37 °C for 2 h. The plates were washed with PBS-T and 4-fold sample dilutions were added to wells. The samples were diluted in 2% skim milk in PBS-T. After incubating at 37 °C for 1.5 h, the plates were washed 5 times with PBS-T. Horse radish peroxidase (HRP)-conjugated anti-pig IgA (AbD Serotec, Raleigh, NC, USA) was added and incubated at 37 °C for 1 h. The plates were washed 5 times and substrate 2,2′-azinobis [3-ethylbenzothiazoline-6-sulfonic acid]-diammonium salt) (ABTS) substrate (KPL, Baltimore, MD, USA) was added. The plates were read at 405 nm after 10 min-incubation at room temperature in the dark. The cut off value was determined as the average of the absorbance of the positive capture of the negative samples + 3 times of the standard deviation. The sensitivity and specificity of the ELISA was calculated based on virus neutralizing (VN) antibody titers as gold standard as 100 and 96.6%, respectively.

Whey was prepared from milk following protocols described earlier [19]. Briefly, skim milk was separated by centrifugation of whole milk or colostrum at 2000 × *g* for 30 min at 4 °C and by collecting the middle portion between the cream layer on top and casein layer in the bottom. Whey was prepared by centrifugation of skim milk at 90 000 × *g* for 60 min at 4 °C. The samples were filtered with 0.45 μm filters and stored at −20 °C.

A plaque reduction virus neutralization assay was performed using the tissue culture-adapted original US PEDV strain PC22A [5]. The protocol was modified slightly from a previously published protocol for transmissible gastroenteritis virus (TGEV) [19]. The sera and whey samples to be tested were heat inactivated for 30 min at 56 °C. The serum or whey samples to be tested were diluted 2-fold and the different dilutions (500 µL) were mixed with an equal volume of 70 plaque forming units (PFU) of the virus. The mixture was incubated at 37 °C for 90 min with gentle rocking. The mixture (500 µL) was then added to duplicate wells of monolayers of Vero cells in 6-well plates that had been washed with serum-free medium. The plates were incubated at 37 °C for 60 min with gentle rocking. The cells were then washed and overlaid with 0.75% low melting point agarose (SeaPlaque, Lonza, Riverside, PA, USA) in serum free media supplemented with tryptose phosphate broth and trypsin as described for the cultivation of PEDV [5]. The plates were incubated in a humid chamber at 37 °C for 3 days. The plaques were stained with 0.001% neutral red solution (Sigma, St. Louis, MO, USA, catalog # N2889). The plaques were counted and the reciprocal of the highest dilution of a serum or whey sample showing an 80% reduction in the number of plaques was defined as its virus neutralization titer.

Mononuclear cells (MNCs) were isolated from the lamina propria of the ileum by using previously described methods [20]. The ELISPOT was done by following procedures as described previously [17]. PEDV PC22A strain-infected (≥80% of cells PEDV antigen positive by cell culture immunofluorescence assay) and acetone-fixed Vero cells in 96-well plates were used as antigens for ELISPOT. The plates were prepared ahead and frozen at −20 °C. Fixed cells were thawed and rehydrated by incubation with RPMI (Life Technologies, Carlsbad, CA, USA) supplemented with 8% fetal bovine serum (FBS) (Atlanta Biologicals, GA, USA) for 5 min at room temperature. Serial dilutions of MNC (5 × 10^3^, 5 × 10^4^ and 5 × 10^5^) were added to duplicate wells of the fixed PEDV PC22A-infected cell monolayers. Plates were centrifuged at low speed (50 × *g*) for 5 min and incubated at 37 °C with 5% CO_2_ overnight. The plates were washed 5 times with PBS-T. Antibody production by the antibody secreting cells (ASCs) was detected by incubating with HRP-conjugated anti-pig IgA (AA140P; AbD Serotec) diluted 1:3000 and added at 100 µL/well or HRP-conjugated anti-pig IgM (Bethyl laboratories, Montgomery, TX, USA) at 1:500 at 100 µL/well or biotinylated anti-pig IgG (KPL, Baltimore, MD, USA) at dilution of 1:20 000 at 100 µL/well and incubated at 1 h at 37 °C. For IgG antibodies, the plates were washed and incubated with HRP-conjugated streptavidin (1:10 000) (Roche, Indianapolis, IN, USA) at room temperature for 1 h. The plates were washed and the spots were developed by adding 3,3′,5,5′-tetramethylbenzidine (TMB) substrate with H_2_O_2_ membrane peroxidase substrate system (KPL) and counted using a light microscope. Counts were averaged from duplicate wells and were expressed relative to 5 × 10^5^ MNC. PEDV specific IgA and VN antibody titers, antibody secreting cell numbers, and virus RNA shedding titers were compared by one-way analysis of variance (ANOVA). Pig diarrhea rates were compared by Fisher’s exact test. A value of *p* < 0.05 was considered statistically significant.

Original US PEDV or S-INDEL Iowa106 PEDV inoculation induced complete and partial protection, respectively against the original US PEDV challenge. Post inoculation, 100% of original US PEDV and S-INDEL Iowa106-inoculated piglets had diarrhea and shed PEDV (Table 1). Mean virus RNA shedding titers were higher during the first and third week post inoculation (15–21 dpi) in the original PEDV-inoculated piglets compared with the S-INDEL Iowa106-inoculated piglets, but no such difference was observed during the second week post-inoculation (Table 1). To examine the development of protective active immunity against the highly virulent PEDV strain, piglets were challenged with the original US PEDV strain. Fecal virus shedding and diarrhea were assessed since they are the major parameters in assessing PEDV infection and the severity of disease as well as protective immunity against the viral strains. Mean virus RNA shedding titers were similarly low and did not differ significantly in both S-INDEL Iowa106 (6.0 ± 3.1 log_10_ GE/mL) and original US PEDV PC21A-inoculated piglets (6.2 ± 0.3 log_10_ GE/mL), but significantly lower than that (8.7 ± 0.8 log10 GE/mL) of the mock (control)-inoculated piglets post-challenge. No pigs (0/4) had diarrhea in the original US PEDV PC21A-inoculated piglets compared with 81% (13/16) in the S-INDEL Iowa106- and 100% (5/5) in the mock (control)-inoculated piglets at 1–7 dpc (Table 1). No gross and microscopic lesions were observed among the pigs [8].

**Table 1:** **Fecal consistency scores and fecal PEDV RNA shedding titers in original US PEDV-, S-INDEL PEDV-, and mock (control)-inoculated piglets post inoculation and after challenge with the original US PEDV virus**

	Mean fecal PEDV RNA shedding titers (SD) (log_10_ GE/mL)			Mean fecal consistency scores (SD)			Pig diarrhea rates (%)		
	Control	S-INDEL	Original US PEDV	Control	S-INDEL	Original US PEDV	Control	S-INDEL	Original US PEDV
DPI 1–7	ND	8.2^b^ (1.1)	9.1^a^ (1.0)	0^c^	1.7^b^ (1.0)	2.4^a^ (0.5)	0^b^	100^a^	100^a^
DPI 8–14	ND	7.8 (1.1)	8.2 (0.2)	0	0.3 (0.5)	0.8 (0.9)	0^b^	0^b^	28^a^
DPI 15–21	ND	6.7^b^ (0.9)	8.1^a^ (0.3)	0	0	0	0	0	0
DPC 1–7	8.7^a^ (0.8)	6.0^b^ (3.1)	6.2^b^ (0.3)	1.2^a^ (1.0)	1.1^a^ (0.9)	0^b^	100^a^	81^a^	0^b^

Fecal viral RNA shedding titers were determined by TaqMan real-time reverse transcription-PCR.

Fecal consistency scores 0, 1, 2 and 3 correspond to normal, pasty, semi-liquid, and liquid feces, respectively, with scores of ≥ 2 corresponding to diarrhea.

Values with different letters (a, b and c) differ significantly between groups (one-way ANOVA for means and Fisher’s Exact test for diarrhea rates, respectively. *p* < 0.05).

SD: standard deviation, ND: not detectable.

Serum antibody responses were lower in piglets inoculated with the S-INDEL Iowa106 strain compared with the original US PEDV. PEDV specific serum virus neutralization antibody titers were significantly lower in the S-INDEL Iowa106 group compared with the original US PEDV-inoculated piglets at pre-challenge (0 dpc) (Figure 1B). Similar to VN titers, PEDV specific IgA antibody titers were also lower in the S-INDEL Iowa106-inoculated, compared with the original US PEDV-inoculated piglets pre-challenge (0 dpc) (Figure 1C). Compared with the S-INDEL Iowa106 inoculated piglets, the original US PEDV inoculation of piglets induced higher and more rapid PEDV specific IgA antibody responses by 7 dpi (Figure 1C).

**Figure 2:**
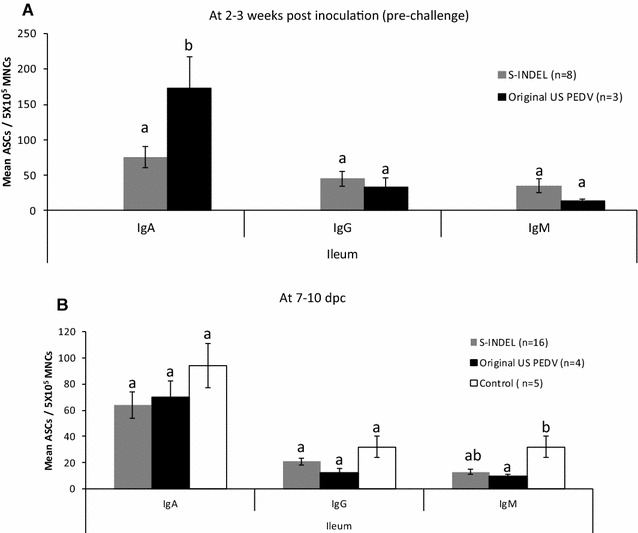
**Isotype-specific antibody-secreting cell (ASC) responses to PEDV in ileum of pigs at 2–3** **weeks post-inoculation (pre-challenge) (A), and at 7–10** **days post challenge (dpc) (B).**

Intestinal PEDV specific ASC responses were assessed to examine whether S-INDEL Iowa106 and original US PEDV PC21A strains induced any differential intestinal antibody responses. The S-INDEL Iowa 106 strain induced lower intestinal PEDV specific IgA ASC responses. Virus specific IgA ASC responses were significantly lower in the S-INDEL Iowa106-inoculated piglets compared with the original US PEDV-inoculated piglets at 0 DPC (Figure 2A). No differences in virus specific IgG and IgM ASC responses were observed between S-INDEL Iowa106 and original US PEDV-inoculated piglets pre-challenge (Figure 2A). Likewise, intestinal isotype specific ASCs responses were similar between S-INDEL Iowa106 and original US PEDV-inoculated piglets post-challenge (28–31 DPI/7–10 DPC) (Figure 2B). Further, the numbers of IgA ASC were consistently higher than the numbers of IgG and IgM ASC for all groups in ileum.

**Figure 3:**
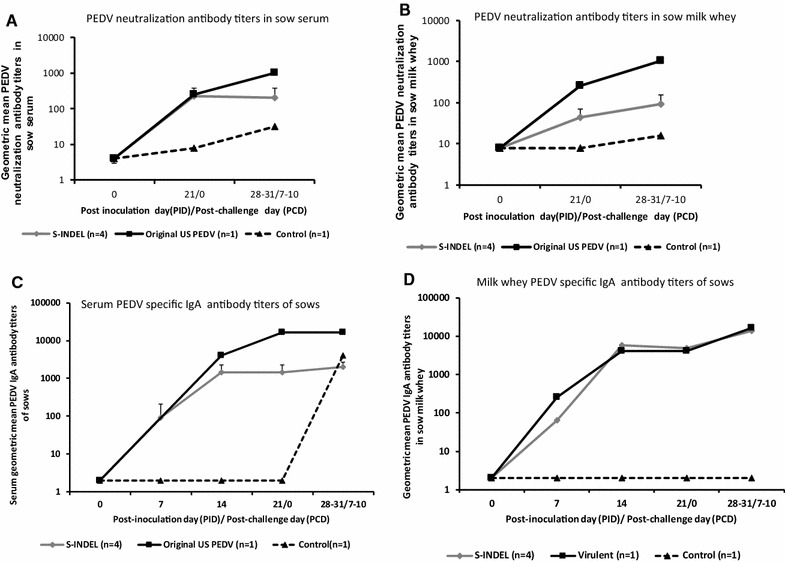
**PEDV specific antibody responses of sows in serum and milk whey.** Sow serum (**A**) and milk whey (**B**) virus neutralization antibody titers were quantified by plaque reduction virus neutralization assay. Sow serum (**C**) and milk whey (**D**) PEDV specific IgA antibody responses were quantified by ELISA.

Contact exposure of sows induced virus specific antibody responses in sows. Inoculation of piglets with PEDV caused contact exposure of sows to virus as indicated by fecal virus RNA shedding and the presence of virus specific antibody titers in whey and serum (Figure 3) [8]. Statistics were not done for antibody titers of sows because of *n* = 1 for two of the groups. The sows of piglets inoculated with the S-INDEL Iowa106 and the original US PEDV strains showed similar virus neutralization antibody responses in the serum pre-challenge (0 dpc) (Figure 3A). However, whey PEDV neutralization antibody titers and serum PEDV IgA antibody titers were consistently lower in sows of the S-INDEL Iowa106-inoculated piglets compared with the sow of the original US PEDV-inoculated piglets (Figures 3B and C). Further, virus specific IgA antibody titers as determined by ELISA in whey were similar between the sows of piglets inoculated with the S-INDEL Iowa106 and the original US PEDV strains (Figure 3D).

## Discussion

The severity of PEDV infection depends mainly on the age of the piglets [21]. Apart from the age, the strain of PEDV also influences viral pathogenesis. Particularly, studies from our laboratory, as well as from other laboratories, showed that S-INDEL PEDV strains cause less severe infection compared with the original PEDV strains [7, 8]. However, whether the differences in severity of infection between those PEDV strains have any impact on eliciting protective immunity is unknown. Our results showed that the S-INDEL Iowa106 induced only partial protective immunity against the original US PEDV strain challenge.

Vaccination is considered as an effective method to control PEDV infection in piglets. Mucosal immunity plays a crucial role in conferring protective immunity against enteric infections. Specifically, mucosal antibody responses are correlates of protection against many enteric viral infections. Thus, studying mucosal immunity, such as intestinal antibodies and ASC responses to PEDV strains reveals important information about protective immunity against PEDV. Similar to other enteric viral infections such as TGE, porcine respiratory coronavirus [22] and rotavirus [23], in this study both of the PEDV strains also induced predominantly IgA ASC responses in the intestine. Further, single exposure of nursing piglets to the original US PEDV strain or S-INDEL Iowa106 strains resulted in induction of intestinal virus specific IgA ASCs in piglets.

Previous studies showed that the S protein of PEDV plays an important role in eliciting protective immunity [24, 25]. Results of this study indicated that mutations in the S protein of S-INDEL Iowa106 strain were associated with reduced systemic and mucosal antibody responses in comparison with responses to the original US PEDV strain. Specifically, ileal virus specific IgA ASCs were significantly lower in the S-INDEL Iowa106-inoculated piglets compared with the original US PEDV-inoculated piglets pre-challenge. However, the original US PEDV and S-INDEL strains induced similar levels of virus specific IgG and IgM ASCs responses in the ileum. These results suggest that IgA ASC, but not IgG or IgM ASCs, played a major role in intestinal immunity against PEDV. Further, the S-INDEL Iowa106 induced IgA ASC responses coincided with the partial protection against original US PEDV challenge, as indicated by the significant reduction in virus RNA shedding titers in S-INDEL-inoculated piglets compared with mock-inoculated piglets. These in vivo results also confirmed our previous in vitro findings in which antisera against S-INDEL Iowa106 neutralized the original US PEDV strain [26]. Similar to our results, sows that were contact exposed to the S-INDEL variant PEDV conferred partial protection to their piglets post challenge with the original US PEDV strain [27].

The milder S-INDEL Iowa106 infection might be a potential reason for a significant reduction in ileal IgA ASC responses in the S-INDEL Iowa106-inoculated piglets compared with the original US PEDV PC21A-inoculated piglets at 14–20 dpi. Further, the original US PEDV PC21A-inoculated, but not the S-INDEL-inoculated piglets were completely protected against diarrhea post-challenge. This might be due to the lower level of intestinal IgA ASCs responses in the S-INDEL Iowa106-inoculated piglets which might be a possible reason for the failure to induce complete protection against diarrhea post-challenge (1–7 dpc). The specific reason for lower virus-specific antibody responses in the S-INDEL-inoculated compared with the original US PEDV-inoculated piglets is unknown. We speculate that differences in replication kinetics between the PEDV strains might have caused the lower antibody responses in the S-INDEL-inoculated piglets [8]. Specifically, we observed restricted viral infection as indicated by lower percentage of virus infected enterocytes in S-INDEL Iowa106-inoculated piglets compared with original US PEDV PC21A piglets at various post-inoculation time points by immunochemistry staining [8]. Further 3 of the 4 litters of the S-INDEL- inoculated piglets had shorter duration of diarrhea compared with the original US PEDV-inoculated piglets (Table 1). These factors likely have an impact on induction of virus specific antibody responses both mucosally and systemically. Our findings are further supported by an earlier study [28] in which inoculation of virulent PEDV induced complete protection but exposure of piglets to an attenuated strain of PEDV elicited only partial protection against challenge. The authors also hypothesized that differences in viral antigen levels in the intestine between the virulent and attenuated PEDV viruses might have caused differences in induction of protective immunity in piglets [28]. However it should be determined whether a booster dose of S-INDEL Iowa106 induces more complete protection against diarrhea. This idea is supported by our observation that virus specific antibody responses reached comparable levels in both S-INDEL Iowa106 and original US PEDV-inoculated piglets post-challenge (Figures 1, 2). Additionally, attenuated TGEV induced lower levels of intestinal virus specific IgA ASCs compared with virulent TGEV in an earlier study [29]. Thus, differences in virus replication kinetics between the S-INDEL Iowa106 and the original US PEDV PC21A strain might be a reason for lower induction of virus specific IgA ASCs in ileum.

Infection of piglets with the selected PEDV strains also induced virus specific IgA antibody responses in sows through contact exposure followed by infection and virus RNA shedding. Further, induction of virus neutralizing antibody titers in milk whey of the sows also showed that S-INDEL Iowa106 can also induce lactogenic antibodies. Among the sows, milk whey virus neutralization antibody titers at 21 dpi/0 dpc were lower in the S-INDEL Iowa106 sows compared with the original US PEDV contact exposed sow. Thus, we speculate that differences in milk whey virus neutralizing antibody titers might also potentially cause differences in protective efficacy between the S-INDEL Iowa106 and original US PEDV-inoculated groups.

In summary, Iowa106 strain of PEDV induced a lower magnitude of antibody responses against the original US PEDV strain in pigs compared with that induced by the original US PEDV strain, and resulted in partial protection against challenge with the original US PEDV strain.

## Abbreviations

S-INDEL: spike insertion and deletion
PEDV: porcine epidemic diarrhea virus
dpc: days post-challenge
dpi: days post-inoculation
ASCs: antibody secreting cells
ELISPOT assay: enzyme-linked immunospot assay
ANOVA: analysis of variance
